# Rashba-Type Band Splitting Effect in 2D (PEA)_2_PbI_4_ Perovskites and Its Impact on Exciton–Phonon Coupling

**DOI:** 10.1021/acs.jpclett.4c01957

**Published:** 2024-07-30

**Authors:** Supriya Ghosh, Bapi Pradhan, Arkamita Bandyopadhyay, Irina Skvortsova, Yiyue Zhang, Christian Sternemann, Michael Paulus, Sara Bals, Johan Hofkens, Khadga J. Karki, Arnulf Materny

**Affiliations:** † School of Science, Constructor University, Campus Ring 1, 28759 Bremen, Germany; ∥ Department of Chemistry and Biochemistry, The Ohio State University, 100 West 18th Avenue, Columbus, Ohio 43210, United States; ○ Electron Microscopy for Materials Research, University of Antwerp, Groenenborgerlaan 171, 2020 Antwerp, Belgium; μ Fakultät Physik/DELTA, Technische Universität Dortmund, 44221 Dortmund, Germany; § Department of Chemistry, KU Leuven, Celestijnenlaan 200F, 3001 Heverlee, Belgium; π Bremen Center for Computational Materials Science, University of Bremen, 28359 Bremen, Germany; δ Max Planck Institute for Polymer Research, Ackermannweg 10, 55128 Mainz, Germany; ‡ Guangdong Technion Israel Institute of Technology, 241 Daxue Road, Shantou, Guangdong Province 515603, P. R. China

## Abstract

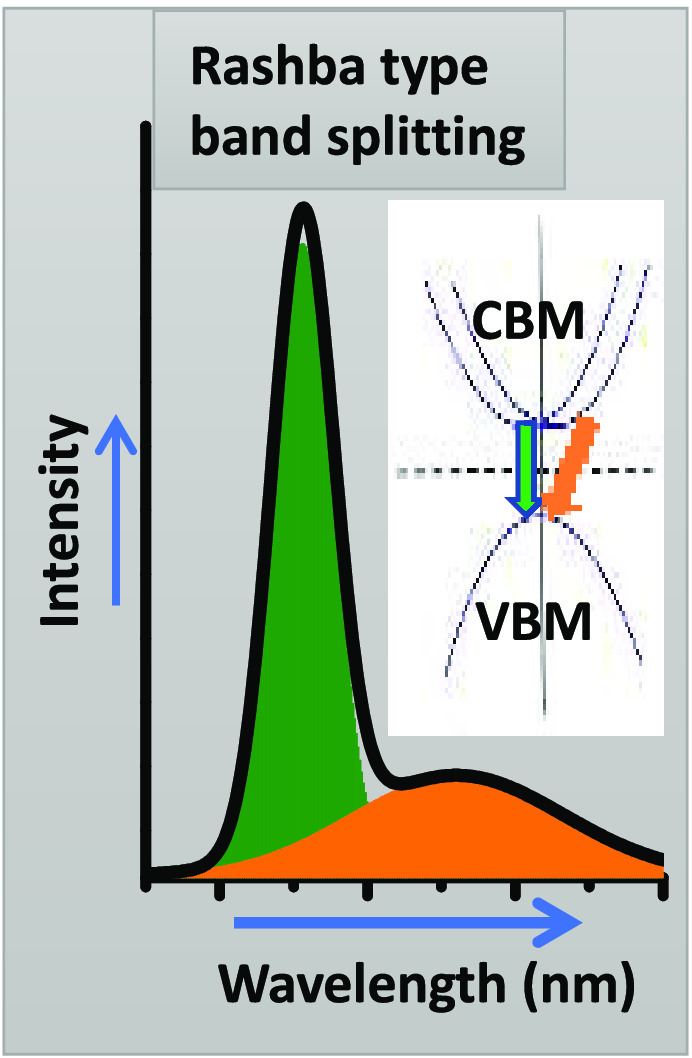

Despite a few recent reports on Rashba effects in two-dimensional (2D) Ruddlesden–Popper (RP) hybrid perovskites, the precise role of organic spacer cations in influencing Rashba band splitting remains unclear. Here, using a combination of temperature-dependent two-photon photoluminescence (2PPL) and time-resolved photoluminescence spectroscopy, alongside density functional theory (DFT) calculations, we contribute to significant insights into the Rashba band splitting found for 2D RP hybrid perovskites. The results demonstrate that the polarity of the organic spacer cation is crucial in inducing structural distortions that lead to Rashba-type band splitting. Our investigations show that the intricate details of the Rashba band splitting occur for organic cations with low polarity but not for more polar ones. Furthermore, we have observed stronger exciton–phonon interactions due to the Rashba-type band splitting effect. These findings clarify the importance of selecting appropriate organic spacer cations to manipulate the electronic properties of 2D perovskites.

In the past few years, metal halide perovskite (MHP) materials have evolved remarkably for optoelectronic applications.^1−6^ Due to their unique structural and electronic properties such as sharp and narrow emission peak, high photoluminescence quantum yield (PLQY), and their facile low-cost synthesis, MHPs have opened new avenues for the future of light-emitting diode technology (LED).^5,7−11^ Considerable efforts have been employed to develop new perovskite materials and innovative device architectures,^3,12−15^ with extensive research devoted to understanding the working mechanisms of these devices.^16−19^ The luminescence efficiency of the devices can be significantly improved by controlling the carrier injections and reducing the nonradiative recombination.^16,20,21^ Low band gap three-dimensional perovskite LEDs have shown promising advancements in external quantum efficiency (EQE), surpassing 20%,^22^ in comparison to organic LEDs (OLEDs).^23^ Despite this significant improvement in efficiency, the development of 3D-perovskite-based LEDs faces challenges related to poor structural and operational stability.^24^ Two factors that reduce stability are well-known. First, it decomposes at high humidity and temperature, due to its soft structure.^25^ Second, under the influence of an electric field, organic cations or halide ions are moved, making undesirable changes in the structures of perovskites.^25^ The key bottleneck is still these factors for prospective industrialization.^26^ One of the potential solutions for these problems is to use two-dimensional (2D) MHPs. It is considered an emerging material for optoelectronic applications with significant promise.^3,27−30^ 2D MHPs can be formed by incorporating long organic cations into the ABX_3_ perovskite frameworks, which do not fit into the octahedral network. Like for the 3D systems, by controlling the organic spacer cations in the 2D materials, the optical properties can be tailored to obtain specific characteristics for various optoelectronic applications.

For applications in light-emitting devices and solar cells, it is very crucial to understand the underlying photophysical processes such as electron recombination, electron trapping, electron–phonon interaction, etc. Electron–phonon interaction is particularly important, as it is related to physical properties that affect the functionality of devices. Like their 3D counterparts, 2D perovskites typically exhibit strong electron–phonon interactions^31−33^ because of the intrinsic ionic nature and softness of the material. Strong electron–phonon interactions enhance the local lattice distortions, leading to the formation of self-trapping excitons (STEs). The localization or self-trapping of photogenerated excitons, resulting in Stokes-shifted broad-band photoluminescence which is useful for applications requiring wide spectral coverage.^34^ However, the assignment of STE to a broad emission with a large Stokes shift is contradicted by a recent work on single 2D perovskite flakes.^35^ The modification of local structures also influences the photophysical properties of 2D perovskites.^17^ Low-energy emission is observed specifically from the high and medium *n*-phases (*n* represents number of layer), whereas high-energy emission comes from lower *n*-phases. Furthermore, electron–phonon coupling within the lattice of lead halide perovskites is crucial for their favorable optoelectronic properties, such as long carrier lifetimes and high diffusion lengths. However, recent findings of Rashba-like spin splitting and indirect band formation necessitate a reconsideration of how these interactions function within the perturbed electronic band structure.^36−38^ The Rashba-like effects in metal halide perovskites arise due to the combination of strong spin–orbit coupling (SOC) from heavy metal atoms such as Pb and the lack of inversion symmetry in their crystal structures. The strong SOC modifies the electronic structure significantly, while the lack of inversion symmetry lifts the spin degeneracy, leading to band splitting.^39^ The study of Rashba-type effects in 2D perovskites holds immense promise for advancement of the fundamental understanding of these materials and their potential applications. A deeper understanding of these effects is crucial for tailoring the electronic properties of perovskites to enhance their performance in optoelectronic and spintronics devices.

In our work presented here, we investigate the different emissive states and the effects of Rashba-type band splitting by using temperature-dependent two-photon PL spectroscopy in combination with band-structure calculations of 2D (PEA)_2_PbI_4_ (HP) and (F-PEA)_2_PbI_4_ (FP) perovskites. In both perovskites, we observed narrowing of emission line widths and intensity enhancements with the lowering of temperature. HP shows two PL peaks at low temperature, whereas FP shows one PL peak throughout the full temperature range. The Rashba-type band splitting effect was found in HP, whereas it could not be observed in FP. This resulted in a stronger exciton–phonon coupling in HP compared with FP. We found that the Rashba-type band splitting effect depends on the polarity of the organic spacer cations.

HAADF-STEM images shown in Figure 1a,d reveal a 2D morphology for both HP and FP samples with a significant difference in shape. HP particles are present in a form of circular sheets, whereas FP consists of square sheets of the same size range as HP (around 200–800 nm). Energy-dispersive X-ray spectroscopy (EDS) results (Figure S1a,b) combined with maps (Figure 1b,e) prove the homogeneity of elemental distribution as well as the composition of the perovskites. We observe a clear fluorine *K*_∝_ peak in the EDS spectrum for the FP sample (Figure S1b). Electron diffraction (ED) patterns for HP along the [011] direction and for FP along the [100] direction are shown in Figure 1c,f, respectively; they confirm the expected crystal structures. HP belongs to the triclinic *P*-1 space group, while for FP the monoclinic *P*_1_21/*c*1 structure is found at room temperature.

**Figure 1:**
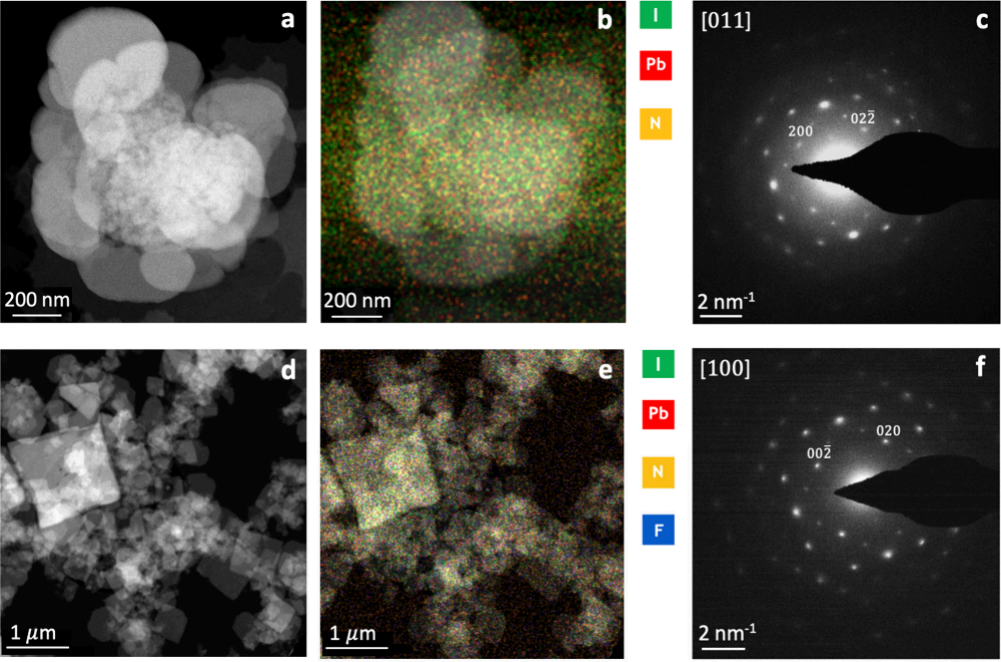
(a and d) HAADF-STEM images, (b and e) color-coded STEM-EDS combined elemental maps with corresponding average compositions, (c and f) ED patterns for (PEA)_2_PbI_4_ (HP) and (F-PEA)_2_PbI_4_ (FP), respectively. For panels b and e, compositions calculated using the Cliff Lorimer method are N 28.3 ± 3.5 at. %, Pb 14.9 ± 0.5 at. %, I 56.8 ± 1.1 at. %, and F 23.2 ± 1.2 at. %, N 21 ± 2 at. %, Pb 11.3 ± 1.7 at. %, I 44.5 ± 2.2 at. %, respectively.

The 2D nature of the material can be corroborated by analyzing the structure and morphology of the samples. To further confirm the crystal phase, we conducted synchrotron grazing incidence wide-angle X-ray scattering (GIWAXS) experiments at room temperature. Figure 2a,b represents the 2D GIWAXS patterns of HP and FP 2D perovskites, respectively, as a function of wave-vector transfer parallel (q_r_) and perpendicular (q_*z*_) to the sample surface. The out-of-plane and in-plane integration peak positions match well with the standard phase of HP and FP (Figures S2 and S3, respectively) in the respective cases.

**Figure 2:**
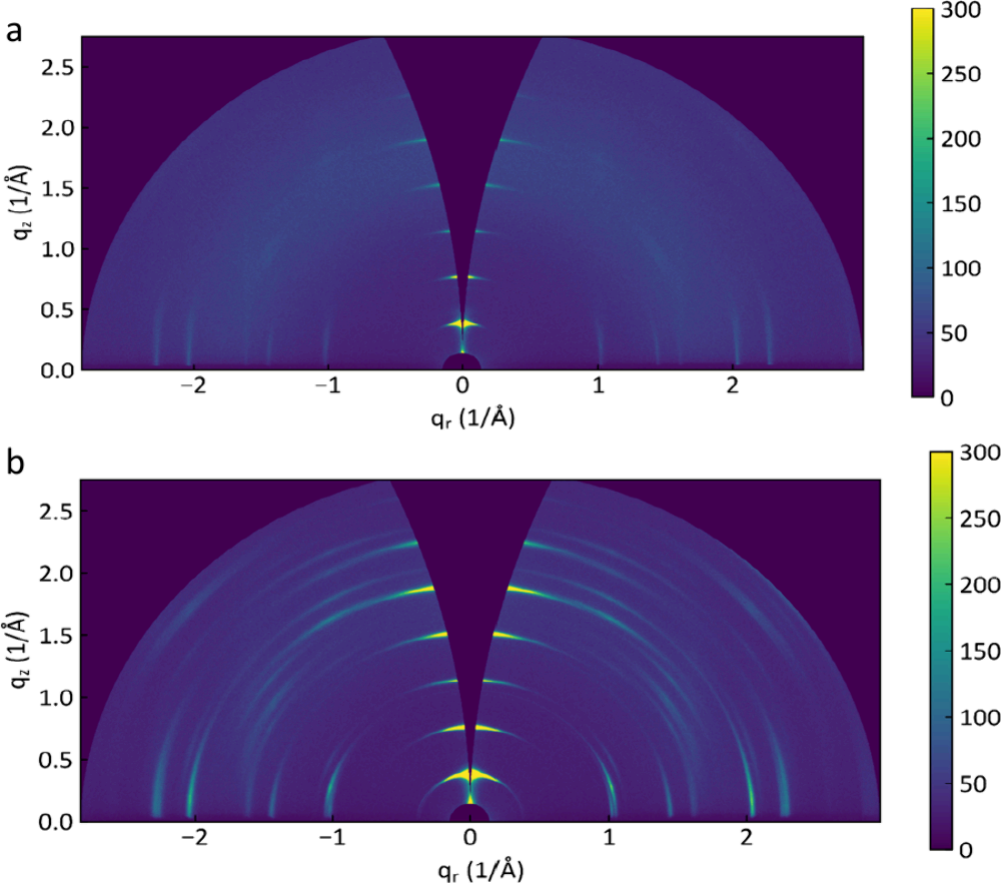
2D GIWAXS scattering patterns of (a) (PEA)_2_PbI_4_ (HP) and (b) (F-PEA)_2_PbI_4_ (FP) measured at 13 keV with an angle of incidence of 0.25°.

Out-of-plane data can be used to fit the (*n*00) and (00*n*) diffraction peaks on a 2θ scale for FPEA and PEA, respectively (Figure S4). These results, analyzed using a Williamson-Hall plot, estimate the strain of the second kind ε to be 0.0059 ± 0.0005 for FP and 0.0041 ± 0.0005 for HP and the crystallite size *L* to be 92 ± 10 nm for FP and 67 ± 10 nm for HP perpendicular to the sample’s surface. However, limitations in accessing broadening due to the grazing-incidence geometry may significantly affect the accuracy of the crystallite size estimation. Therefore, *L* should be regarded only as a minimum size of the flakes’ crystallites in the direction perpendicular to the surface. By examining the (*n*00) and (00*n*) diffraction peaks of FP and HP in the azimuthal direction and assuming isotropic tilt variation, the width of the tilt-distribution for the flakes is estimated. Specifically, this analysis suggests a tilt-distribution width of 11.5° for HP and 15.3° for FP. This finding indicates that HP and FP flakes exhibit different tilt variations and crystal ordering relative to the surface normal, which affects the photophysical properties of HP and FP films. To obtain more detailed information on the systems, we have investigated the temperature-dependent two-photon photoluminescence (2PPL) spectra of the thin films of FP and HP; the results are shown in Figure 3a,b and 3c,d, respectively.

**Figure 3:**
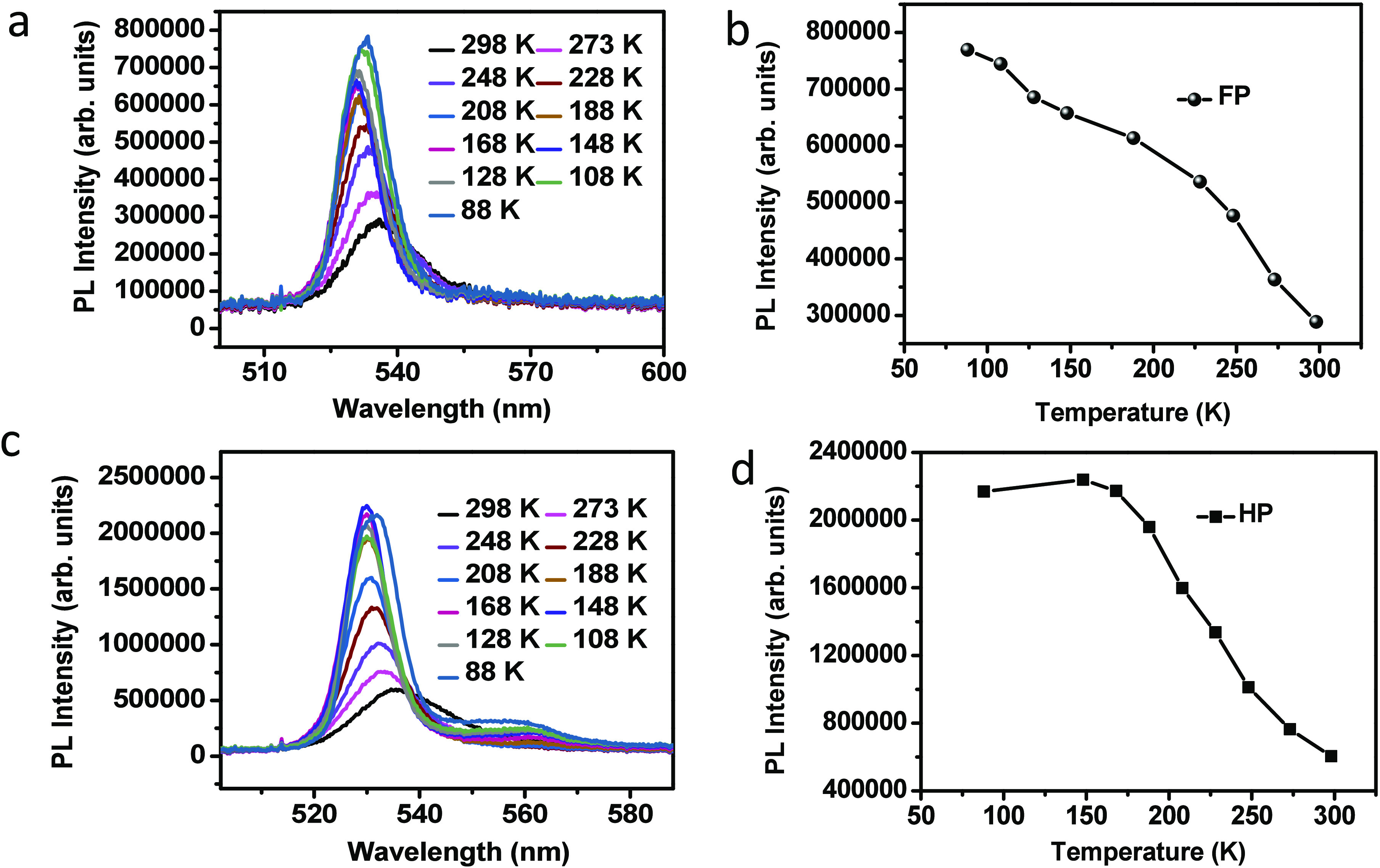
Temperature-dependent photoluminescence spectra of (a) (F-PEA)_2_PbI_4_ (FP) and (c) (PEA)_2_PbI_4_ (HP). Change of PL intensity with temperature observed for FP and HP are shown in panels b and d, respectively.

The sample is cooled at a controlled rate of 3 K/min, starting from room temperature (298 K) down to liquid nitrogen temperature (80 K). At each selected temperature, the spectra are averaged over 20 different points. Overall, a gradual blue shift in the spectra is observed as the temperature decreased. Figure 3b,d shows that the intensity of the spectrum increases with decreasing temperature for both perovskites. We have noticed that the ratio of intensity enhancement between room temperature and low temperature is higher in HP than in FP. These observations indicate that HP shows a substantial reduction of the nonradiative recombination pathways at low temperature. Stranks and co-workers^40^ reported that the PL intensity enhancement is inversely related to the trap density contributing to nonradiative processes. Guo et al.^41^ observed a 72-fold enhancement of the PL intensity with applied pressure in 2D perovskite, which pointed to a reduction of trap states contributing to nonradiative processes. Furthermore, Ramirez et al.^42^ studied the power- and temperature-dependent photoluminescence properties of 2D perovskites. The PLQY enhancement with power and temperature is correlated to lower charge carrier trapping. Based on these findings, we conclude that HP possesses a higher density of nonradiative trap states than FP.^43^ This difference between the two compounds may be due to different lattice distortions caused by the organic cations.^33^ Recently, Liang et al.^44^ reported that trap density and lattice distortions are directly correlated in 2D perovskites. They found that the higher trap density of 2D (*n*-BA)_2_(EA)_2_Pb_3_I_10_ is due to the more severe lattice distortion compared to 2D (*n*-BA)_2_(MA)_2_Pb_3_I_10_. Ni et al.^33^ also described that lattice distortions and increasing number of trapped excitons are correlated. In the case of HP, after 148 K the spectrum starts to shift to the red with a prominent lower-energy peak (LEP) around 560 nm. The red shift after 148 K is attributed to phase transitions from a high-energy phase to a low-energy phase. A similar red shift was previously observed by Ni et al.^33^ in 2D hexylammonium lead iodide (CH_3_(CH_2_)_5_NH_3_)_2_PbI_4_ perovskites at ∼130 K. However, the higher extent of red shift in FP still must be explained; we attribute it to the effect of different organic cations, as the phase-transition behavior depends upon the nature of organic cations in 2D perovskites. Ni and co-workers^33^ confirm this, observing different phase transitions in 2D perovskites with the two different organic cations butylammonium and hexylammonium. It is interesting to note that no LEP is observed in the FP sample. According to previous reports in the literature, the LEP can originate for several reasons. For example, Blancon et al.^45^ observed the LEP below the band gap. These lower-energy states are associated with the local intrinsic electronic structure at the band edges of the 2D exfoliated crystal. However, we exclude that possibility, as in such a case the LEP should be present at room temperatures and should not grow with decreasing temperature. Wu et al.^46^ postulated that the evolution of the broad LEP with lowering of the temperature is associated with trap states that originate from self-trapping of band-edge excitons. We also exclude this possibility, as in this case the emission due to self-trapping of excitons (LEP) should increase and emission due to band edge excitons (which is assigned to a high-energy peak (HEP)) should decrease when the temperature is lowered. With lowering of temperature, self-trapped excitons could not be thermally activated into the band-edge excitons; as a result, HEP should decrease and LEP should increase and vice versa with increasing temperature. Furthermore, the Stokes shift between HEP and LEP should be smaller, as 2D system possess lower activation energy for self-trapping,^46,47^ which is not the case. Kagan and co-workers^48,49^ observed the splitting of the PL and absorbance bands below 75 K; the peaks are equally spaced with 40–43 meV energy gaps, which is consistent with the electron–phonon coupling involving phonons located at organic cations. When we measured the PL at temperatures below 88 K, we did not observe these equally spaced modes. Motti et al.^50^ reported the splitting of the PL band at low temperature. They observed the free carrier population throughout the temperature range from 295 to 4 K. At low temperature (4 K), the PL peak at ∼2.36 eV is assigned to free carrier recombination, and the peak at ∼2.31 eV is explained by exciton recombination. However, in our study, we observe two PL peaks at low temperature (88 K) at ∼2.32 and ∼2.21 eV for the HEP and LEP, respectively. The observed HEP is very close to the PL peak at ∼2.31 eV, which was assigned to the band-edge exciton recombination.^50^ Therefore, we assign the HEP to direct band-edge exciton recombination, whereas the LEP at 2.21 eV is attributed to some indirect carrier recombination below the bandgap. Efros and co-workers^51^ studied the temperature-dependent PL in CsPbBr_3_ perovskite, and there, the observed low-energy PL peak below the optical band gap originates from an exciton sublevel caused by Rashba splitting. Wu et al.^52^ also described that indirect tail states below the direct band gap states arise from a dynamical Rashba-type band splitting effect. Recently, Lafalce et al.^53^ also observed the lower-energy two-photon PL peak below the optical band gap that originates from the Rashba splitting. Steele et al.^54^ described that at low temperature the emission from the low-energy band (Rashba band) increases due to depopulation of high-energy radiative excitons and the stronger contribution of nonradiative decay channels. As the population of optical phonons decreases at low temperature, the thermally induced intersystem crossing between low- and high-energy singlet states is reduced. As a result, more intense low-energy emission is favored. Large spin–orbit coupling (SOC) due to the presence of the heavy metal ion and the absence of a center of inversion gives rise to the Rashba-type effect. Rashba splitting is very likely to occur in MHP; however, the question of whether an inversion symmetry exists or not must be answered.

We assume that the LEP arises from the Rashba-type band splitting effect. To further confirm the presence of Rashba-type band splitting, we calculated the band structure of both perovskites. Using density functional theory (DFT), we learn more about the Rashba band splitting of the 2D Ruddlesden–Popper (RP) HP (Figure 4a) and FP (Figure 4b) perovskites. We have calculated the projected density of states (pDOS) of HP (Figure 4c) and FP (Figure 4d) as the heavier atoms (i.e., I and Pb atoms) contribute more to the SOC. The Rashba effect is prominent in HP because it has a noncentrosymmetric *P*1 space group at low temperature, whereas the FP has a *P*21*c* space group, which is centrosymmetric. Our calculations indicate that the substantial structural distortions caused by different surface terminations are responsible for the observed Rashba effect in HP perovskites. The Rashba splitting is observed around the Γ point of the band structure. The calculated dissociation energies of both systems (i.e., the energies, which are required to break the structures to the most stable components) show that the fluorinated structure is stabilized by an energy of 0.122 eV/atom because of the change of symmetry. The Rashba splitting energy is found to be 0.02 eV.

**Figure 4:**
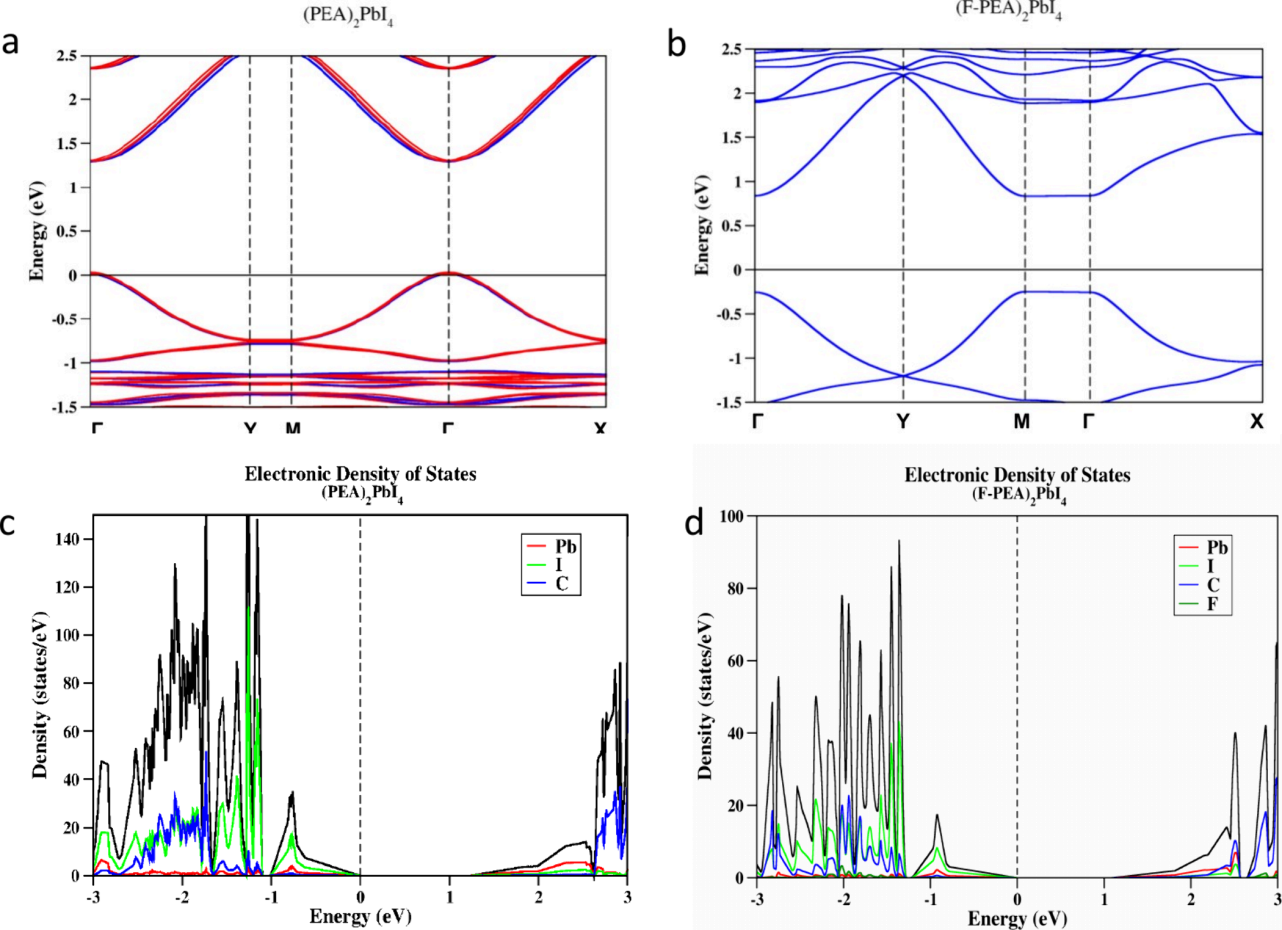
(a and b) Band structures as well as (c and d) projected density-of-states (pDOS) plots for (PEA)_2_PbI_4_ (HP) and (F-PEA)_2_PbI_4_ (FP), respectively.

To further understand the PL dynamics, we carried out time-resolved PL spectral measurements of both samples at room temperature. The decay kinetics shown in Figure 5 is fitted using triexponential functions. All fitting components are given in Table S1. The first component with a time constant of 0.06 ns is attributed to the instrument response function; the second component with 0.6 ns is attributed to the band-edge electron–hole recombination. The third component with 3.4 ns is attributed to carrier recombination through an indirect band gap (lower-energy band). The average lifetimes for HP and FP are 1.51 and 1.04 ns, respectively. The longer lifetime found for HP indicates that their charge carrier recombination occurs via an indirect band gap due to the Rashba-type band splitting effect, which further supports our observation of indirect transitions in HP. However, we have observed very small average lifetime differences only. This could be because most recombination processes involve band-edge carriers at room temperature, and only a very small number of carriers recombine through indirect band states in HP (Table S1). Lafalce et al.^53^ also pointed out that in comparison to low temperature, at room temperature the Rashba effect is very small. PL kinetics measurement at low temperature would give more accurate results as there the carrier recombination through the indirect band gap dominates, as we observed in Figure 3c for HP.

**Figure 5:**
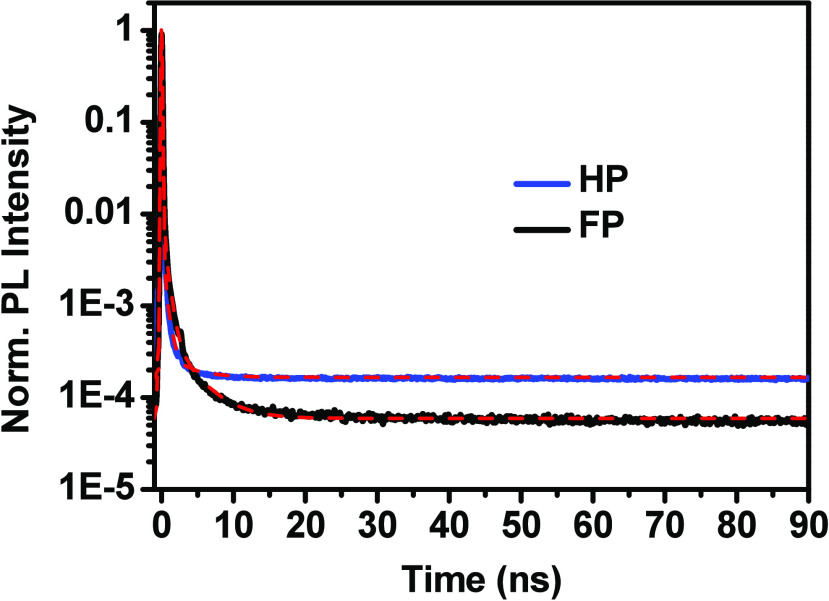
Time-resolved emission kinetics of (PEA)_2_PbI_4_ (HP, blue) and (F-PEA)_2_PbI_4_ (FP, black). The fitting results are shown as dashed red lines.

Next, we investigated how Rashba splitting affects exciton–phonon coupling. Analyzing temperature-dependent emission line width broadening has long been a method to understand carrier–phonon interactions. Phonon scattering in perovskite can be primarily attributed to two processes: scattering from acoustic phonons and scattering from longitudinal optical (LO) phonons, also known as Fröhlich scattering.^33,54^ The symmetric PL line width broadening in HP and FP is attributed to phonon–exciton interactions, as the PL primarily results from exciton recombination. At high temperatures, the interaction between excitons and LO phonons becomes dominant, whereas at low temperatures (below 100 K), exciton–acoustic phonon interactions become more significant, as acoustic phonons, which have lower energies, are more prominent at these lower temperatures.^55^

Previously, Steele et al.^54^ investigated the role of electron–phonon coupling in the evolution of a Rashba-like low-energy emission band in bulk lead halide perovskites. The indirect tail states enhance the electron–phonon coupling and lead to a broad low-energy emission band.^54^ Assessing the temperature-dependent broadening of the emission lines in Figure 6a,b shows that the intrinsic fwhm’s of both perovskites become significantly narrower with decreasing temperature. In Figure 6c, the low-energy band from HP narrows faster than the high-energy band with decreasing temperature, suggesting enhanced optical phonon scattering in this band.

**Figure 6:**
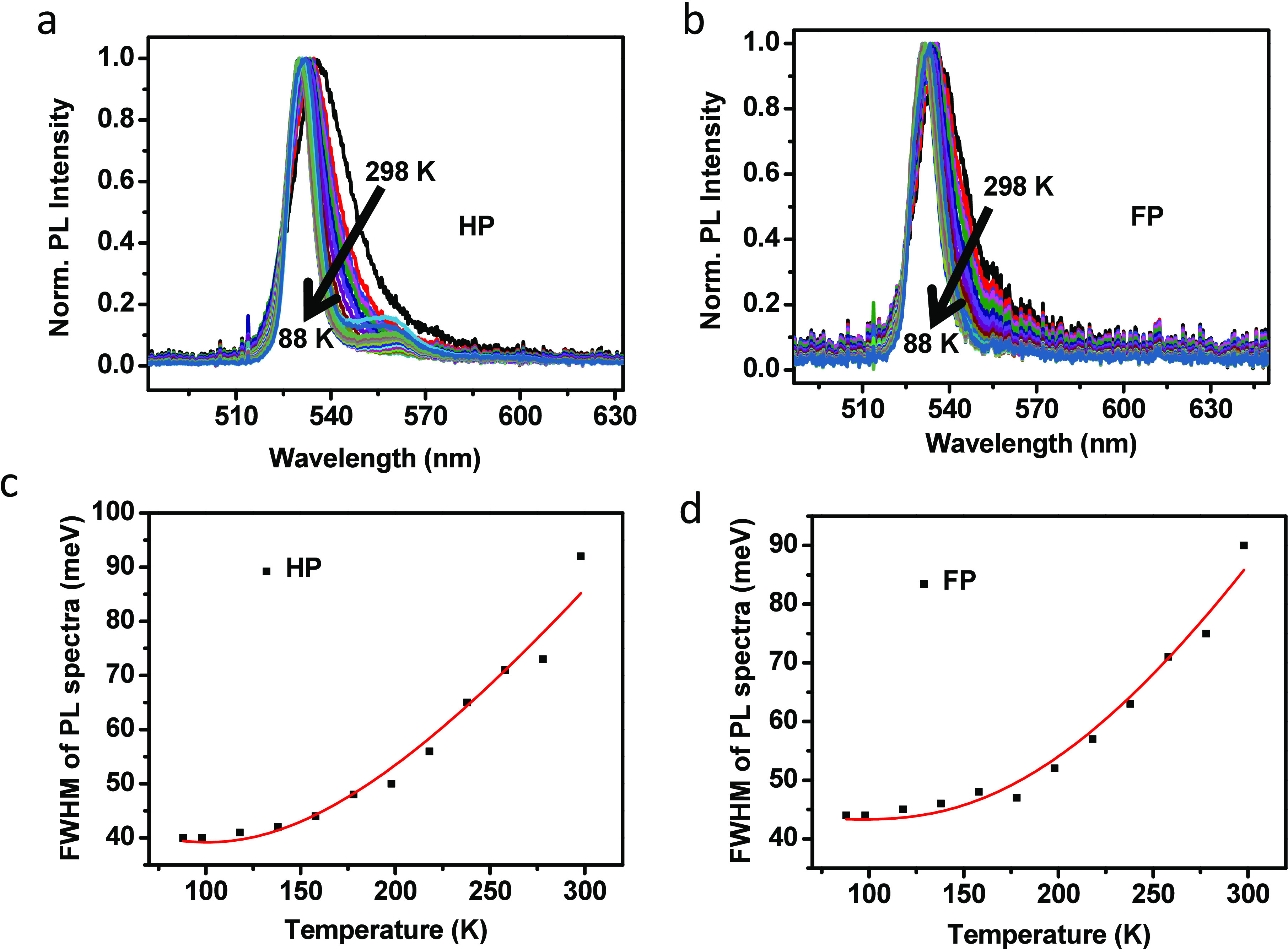
(a and b) Temperature-dependent PL spectra of (PEA)_2_PbI_4_ (HP) and (F-PEA)_2_PbI_4_ (FP), respectively. (c and d) Temperature-dependent PL fwhm of the HP and FP, respectively. The red lines show the fitted line with eq 1, describing the line-width broadening and exciton–phonon coupling to the acoustic phonons at different temperature regime.

By understanding the contributions from different phonon interactions and impurity scattering, we can analyze the temperature-dependent excitonic behaviors. The following equation can be used to understand the relationship between exciton phonon scattering and temperature.^33,55^
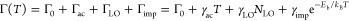
1where Γ_0_ is the inhomogeneous broadening term which is temperature-independent, Γ_ac_ is the homogeneous broadening term describing the contribution of acoustic phonon interactions, and Γ_LO_ is the homogeneous broadening term describing the contribution of LO phonon interactions. γ_ac_ and γ_LO_ are the phonon-coupling strengths for acoustic and LO phonons, respectively. The contribution from LO phonons follows the Bose–Einstein distribution *N*_LO_(*T*) = 1/[e^*E*_LO_/*k*_B_*T*^ – 1]. The term *E*_LO_ represents the energy related to dispersive optical phonons, and *k*_B_*T* represents the thermal energy. At room temperature, acoustic phonon energy is much less than the thermal energy, and the scattering contribution of acoustic phonons is assumed to have a linear dependence on temperature. Γ_imp_ represents the inhomogeneous broadening of the PL line width. This may arises due to the scattering from ionic impurities with an average binding energy *E*_b_ where γ_imp_ is the contribution of impurity scattering to line width broadening. It is observed that the contribution of impurity scattering in perovskite is insignificant.^33,55^ Therefore, it is often assumed as Γ_imp_ ≈ 0 for the analysis. The population of LO phonons increases with temperature, and their contribution at temperatures below 100 K is relatively low. As a result, in this regime, the homogeneous broadening due to exciton–acoustic phonon interactions becomes more significant. Careful investigations in both the low- and high-temperature regimes make it possible to estimate the contribution of optical and acoustic phonons. To quantify both optical and acoustic phonon coupling, the data was fitted using eq 1. The estimated γ_ac_ (acoustic phonon coupling strength) is 0.09 ± 0.2 meV/K for HP and 0.11 ± 0.1 meV/K for FP perovskites. However, to investigate the acoustic phonon scattering with more accuracy, further measurements up to liquid helium temperature (4.2 K) need to be performed. With increasing temperature, the population of LO phonons increases, leading to increase of LO phonon scattering and broadening the PL line width. The estimated γ_LO_ (LO-phonon coupling strength) of HP is with 57 ± 2 meV higher than the 53 ± 2 meV found for FP (Table 1 summarizes the fitting results).

**Table 1:** Parameters Obtained from the Fitting of the PL Line Width Profile with Temperature of (PEA)_2_PbI_4_ (HP) and (F-PEA)_2_PbI_4_ (FP)^a^

Position	Γ_0_ (meV)	γ_LO_ (meV)	γ_ac_ (meV/K)
HP	45 ± 1	57 ± 2	0.09 ± 0.2
FP	41 ± 1	53 ± 1	0.11 ± 0.1

a Compare Figure 6 using eq 1.

The higher coupling strength observed in HP perovskites is primarily due to the strong exciton confinement effect, resulting from the Rashba effect, leading to stronger interactions with phonons.^40,41,54^ The origin of this behavior can be explained by the higher effective mass of carriers within the Rashba split bands.^56,57^ Steele et al.^54^ reported that the effective mass of carriers in the Rashba split band evolves as a function of spin–orbit coupling. According to the Feynman polaron model,^58^ the effective mass has a direct correlation with optical phonon scattering; the carriers with higher effective mass result in more pronounced optical phonon scattering.^54,56,57^

In summary, we have investigated the octahedral distortions and Rashba splitting in the 2D Ruddlesden–Popper (RP) perovskites (PEA)_2_PbI_4_ (HP) and (F-PEA)_2_PbI_4_ (FP) by combining temperature-dependent two-photon PL and time-resolved PL spectroscopy with DFT calculations. We have shown that the origin of the lower-energy PL band in HP is due to Rashba-type band splitting, a feature which is experimentally supported by the longer PL lifetimes observed for HP compared to FP. Furthermore, our theoretical calculation shows that structural distortions or symmetry breaking is the origin of the Rashba-type band splitting effect in HP. In contrast to HP, the presence of a polar organic cation does not lead to structural distortions or symmetry breaking in FP. Furthermore, the Rashba splitting effect leads to higher exciton–phonon coupling in HP than in FP. This study demonstrates the critical role of octahedral distortions and Rashba splitting in determining the photophysical properties of 2D RP perovskites. By manipulation of the organic cation, desired electronic properties can be achieved, making these materials promising candidates for future spintronic devices.

## Supporting Information Available

Experimental methods, XRD patterns, fittings of widths of XRD peaks, EDS spectra, and TEM images (PDF)
