# Supplementary material for: Rashba-Type Band Splitting Effect in 2D (PEA)2PbI4 Perovskites and Its Impact on Exciton–Phonon Coupling
Source: J Phys Chem Lett. 2024 Jul 30;15(31):7970–8. doi: 10.1021/acs.jpclett.4c01957 (PMC11318034; doi:10.1021/acs.jpclett.4c01957)
Supplement: Supplementary file 1 — jz4c01957_si_001.pdf [file jz4c01957_si_001.pdf]

Rashba-Type Band Splitting Effect in 2D $(PEA)_2PbI_4$ Perovskites and Its Impact on Exciton-Phonon Coupling

Supriya Ghosh[†,ǁ,#*], Bapi Pradhan[§,#], Arkamita Bandyopadhyay[π], Irina Skvortsova[^], Yiyue Zhang[§], Christian Sternemann[µ], Michael Paulus[µ], Sara Bals[^], Johan Hofkens[§,δ], Khadga J. Karki [‡*], and Arnulf Materny [†*]

[†]School of Science, Constructor University, Campus Ring 1, 28759 Bremen, Germany

[ǁ]Department of Chemistry and Biochemistry, The Ohio State University, 100 West 18th Avenue, Columbus, Ohio 43210, USA

^Electron Microscopy for Materials Research, University of Antwerp, Groenenborgerlaan 171, 2020 Antwerp, Belgium

[µ]Fakultät Physik/DELTA, Technische Universität Dortmund, 44221 Dortmund, Germany

[§]Department of Chemistry, KU Leuven, Celestijnenlaan 200F, 3001 Heverlee, Belgium

[π] Bremen Center for Computational Materials Science, University of Bremen, 28359 Bremen, Germany

[δ]Max Planck Institute for Polymer Research, Ackermannweg 10, 55128 Mainz, Germany

[‡]Guangdong Technion Israel Institute of Technology, 241 Daxue Road, Shantou, Guangdong Province 515603, P. R. China

[#]Equal contribution

AUTHOR INFORMATION

Corresponding Authors

*E-Mail: ghosh.270@osu.edu

Phone: +1 (0)614 3604134

*E-Mail: khadga.karki@gtiit.edu.cn

Phone: +86 (0)754 88077128

*E-Mail: amaterny@constructor.university

Phone: +49 (0)421 200-3231

Experimental Section

Spectroscopic Techniques

All optical measurements were performed on thin films of HP and FP. These films were prepared by spin coating on a clean ozonized coverslip at 1000 rpm for 30 seconds.

Two-Photon Photoluminescence. The setup has been described elsewhere.[1-2] In brief, a mode-locked Ti-Sapphire oscillator (Synergy from Femtolasers) was used to generate laser pulses with a pulse duration of about 10 fs, a repetition rate of 80 MHz, and a central wavelength of 790 nm. The output of the laser was directed to an inverted microscope. A dichroic mirror (FF670-sdi01−25 × 36, Semrock) was used to separate the excitation beam from the PL. To further reduce the scattered light of the excitation beam, a short pass filter (OD4, Edmund optics, no. 84-698) with a cut-off wavelength of 625 nm was placed before the spectrometer. A reflective objective (36X/0.5 NA, Edmund optics, part 83684410) was used to focus the excitation beam to the sample. The PL was recorded by a spectrometer with a thermoelectrically cooled CCD detector. The sample was placed in a temperature-controlled stage (Linkam Scientific Instruments, LTS420E-P). The temperature was measured with a platinum resistor sensor in close distance to the surface. A temperature control system was used to change the temperature in a range between 80 and 290 K with a linear cooling rate of 3 K/min.

Time-Resolved PL. The lifetimes of the thin-films were probed using a confocal fluorescence microscope (Leica TCS SP8 X). A 470 nm laser with 2.5 MHz repetition rate of 50 ps pulses was employed for the investigation of the FP and HP films. The PL was detected using a fast hybrid photodiode and the exponential decays were fitted with the inbuilt software.

Microscopic Techniques

Transmission Electron Microscopy. High-angle annular dark-field scanning transmission electron microscopy (HAADF-STEM) images, selected area electron diffraction (SAED) patterns and energy-dispersive X-ray spectra in STEM mode (STEM-EDS) were acquired

using a Thermo Fisher Titan Themis Z microscope operating at 300 kV supplied with Super-X EDS system. Acquisition time for EDS measurements was around 500 s. Samples were prepared by dripping a diluted sample solution onto a carbon-coated polymer film copper/gold grid.

Grazing-Incidence Wide-Angle X-ray Scattering (GIWAXS). We performed grazing incidence X-ray diffraction at beamline BL9 of the DELTA synchrotron radiation source (Dortmund, Germany).[3] The 2D diffraction images were collected at an incident energy of 13 keV with a beam size of 0.05 × 1.0 mm^2 (v × h) using a MAR345 image plate detector. The angle of incidence was set to 0.25° and the setup was calibrated with a CeO_2 powder sample as standard. The 2D diffraction images were converted to reciprocal space and analyzed exploiting the program package pygix/pyFAI.[4] The integrations were performed in the azimuthal angular range at 80°±10° and 10°±10° in order to calculate the in-plane and out-of-plane diffraction patterns. In out-of-plane direction the (n00) and (00n) reflections were used to determine the lower limit of the crystallite size in this direction as well as the strain of the second kind for $(F\text{-}PEA)_2PbI_4$ and $(PEA)_2PbI_4$. Moreover, the widths of the (n00) and (00n) diffraction peaks in azimuthal direction were analyzed to determine the tilt-distribution of the flakes.

Computational Methods

We calculated the electronic properties of HP and FP using spin-polarized density functional theory (DFT) as implemented in the Vienna ab initio Simulation Package (VASP).[5-7] The exchange correlation interactions were treated with the Perdew–Burke–Ernzerhof (PBE) formulation of the generalized gradient approximation (GGA).[8] We used a full-potential projected plane-wave framework with a cut-off energy of 350 eV for the plane-wave basis set. We have used ultrasoft pseudopotentials with 14, 7, 7, 4,5 and 1 electrons for Pb, I, F, C, N and H atoms, respectively. The experimental cif files are used to generate the structures. Additionally, we have applied the tetrahedron method with Blöchl corrections for smearing while optimizing the structures. The structures are optimized until the force per atom is less than -0.02 eV. For band structures, we performed spin-orbit coupling (SOC) corrected calculations.

Material Synthesis

0.8 mmol of phenylethylammonium iodide (PEAI, Greatcellsolarmaterials) or 4-Fluoro-Phenylammonium iodide (4F-PEAI, Greatcellsolarmaterials) and 0.4 of mmol PbI_2 (Sigma, 99%) were dissolved in a mixture of 10 mL of DMF and 12.5 µL of *n*-octylamine to form a perovskite precursor solution. Then, 15 µL of the perovskite precursor solution was quickly dropped into 10 mL of toluene under vigorous stirring. After 30 min, the solution was centrifuged at 3,500 rpm for 5 min, and the precipitates were dispersed in hexane for further use.

Fitting Parameters

Table S1. Fitting parameters determined from the PL kinetics of FP and HP perovskites.

**Sample**	**τ_1 (ns)***	**τ_2 (ns)**	**τ_3 (ns)**	**$\tau_{average}$ (ns)**
FP	0.06<br>(100%)	0.6<br>(85%)	3.4<br>(15%)	1.04
HP	0.06<br>(100%)	0.6<br>(67%)	3.4<br>(33%)	1.51

***τ_1 (ns) is due to the instrument response function**

Energy-Dispersive X-Ray Spectroscopy (EDS)

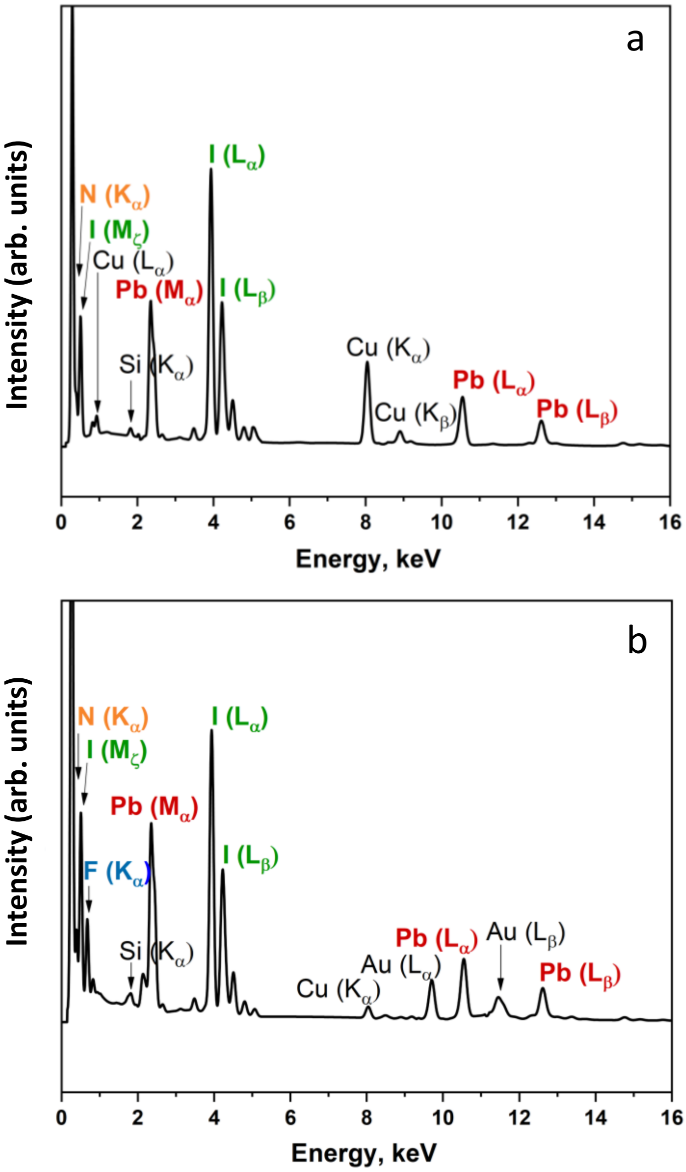

Figure S1. EDS spectra of a) $(PEA)_2PbI_4$ and b) $(F\text{-}PEA)_2PbI_4$. Cu and Au signals emerge from the sample holder, while Si signal originates from the EDS detection system.

Compilation of HP and FP Diffraction Results

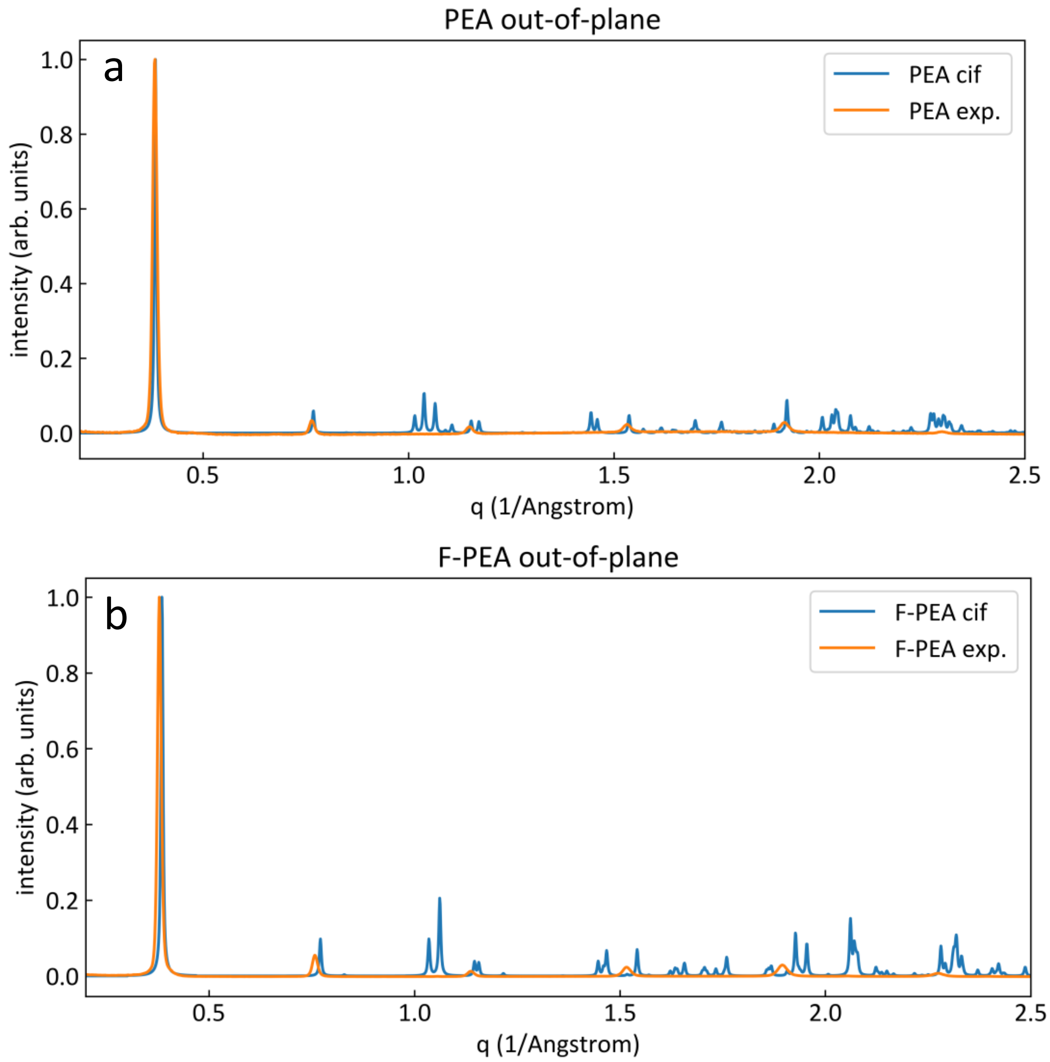

Figure S2. Integrated out-of-plane diffraction patterns of a), b) PEA (HP), and c), d) F-PEA (FP).

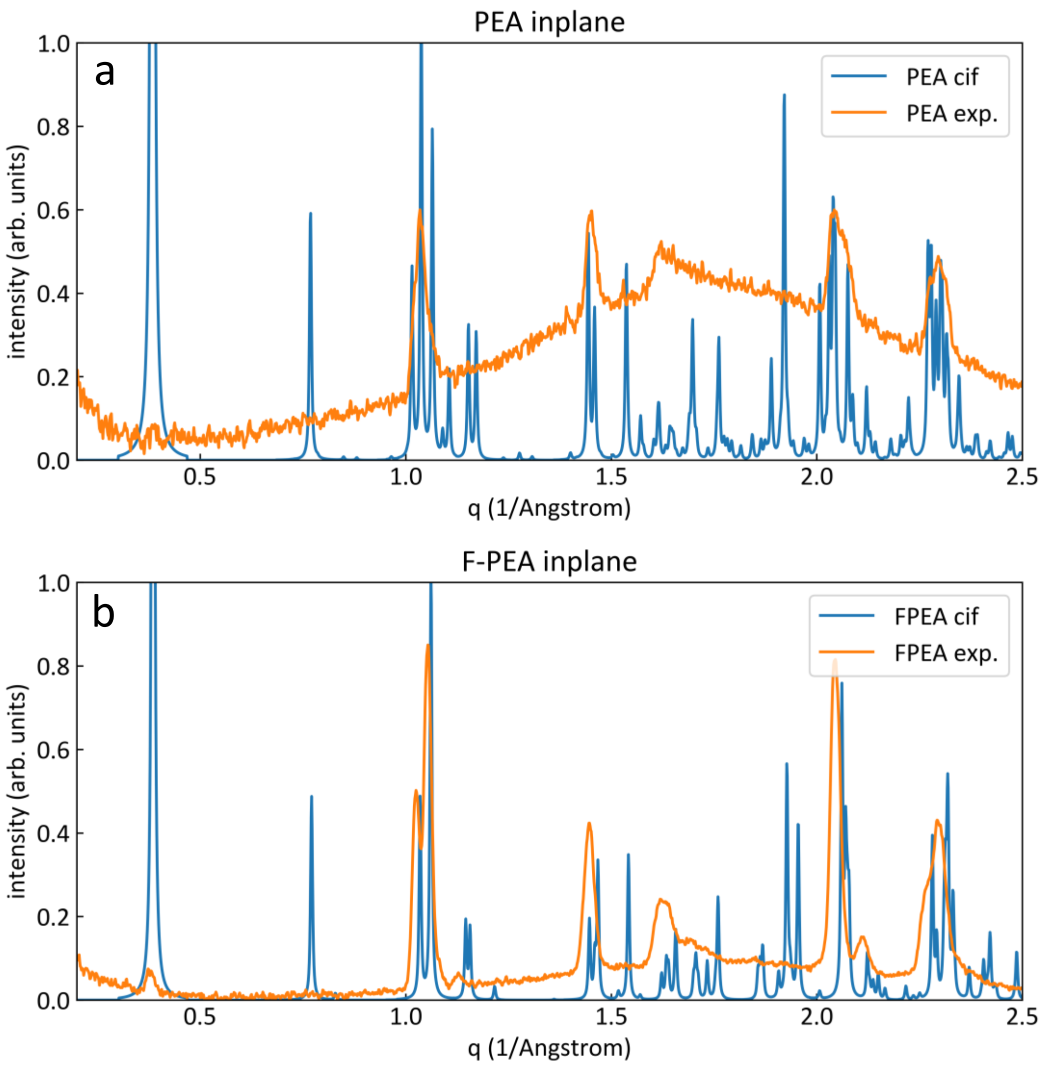

Figure S3. Integrated in-plane diffraction patterns of a) PEA (HP), and b) FPEA (FP).

Fitting of the Widths of the (n00) and (00n) Diffraction Peaks

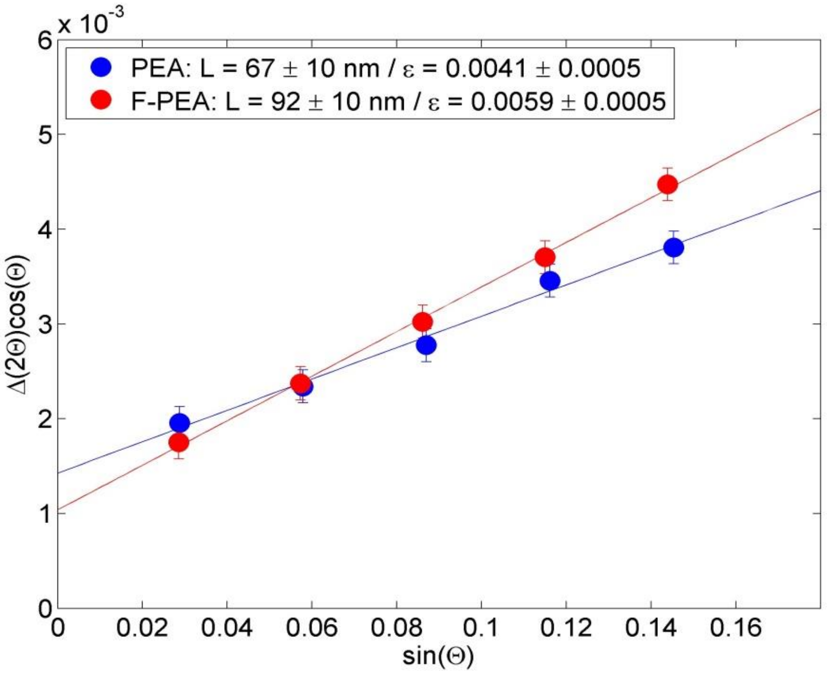

Figure S4. Williamson-Hall plot using the widths $\Delta\big((2\theta)\big)$ of the (00n) and (n00) out-of-plane diffraction peaks for PEA (HP) and F-PEA (FP), respectively.

References

1. Ghosh, S.; Pradhan, B.; Zhang, Y.; Hofkens, J.; Karki, K. J.; Materny, A., Nature of the Different Emissive States and Strong Exciton–Phonon Couplings in Quasi-Two-Dimensional Perovskites Derived from Phase-Modulated Two-Photon Micro-Photoluminescence Spectroscopy. *Phys. Chem. Chem. Phys.* **2021**, *23*, 3983-3992.
2. Kumar, P.; Shi, Q.; Karki, K. J., Enhanced Radiative Recombination of Excitons and Free Charges Due to Local Deformations in the Band Structure of MAPbBr3 Perovskite Crystals. *J. Phys. Chem. C* **2019**, *123*, 13444-13450.
3. Krywka, C.; Sternemann, C.; Paulus, M.; Javid, N.; Winter, R.; Al-Sawalmih, A.; Yi, S.; Raabe, D.; Tolan, M., The Small-Angle and Wide-Angle X-Ray Scattering Set-up at Beamline Bl9 of Delta. *J. Synchrotron Radiat.* **2007**, *14*, 244-251.
4. Kieffer, J.; Valls, V.; Blanc, N.; Hennig, C., New Tools for Calibrating Diffraction Setups. *J. Synchrotron Radiat.* **2020**, *27*, 558-566.
5. Kresse, G.; Hafner, J., Ab Initio Molecular Dynamics for Liquid Metals. *Phys. Rev. B* **1993**, *47*, 558.
6. Kresse, G.; Furthmüller, J., Efficiency of Ab-Initio Total Energy Calculations for Metals and Semiconductors Using a Plane-Wave Basis Set. *Comput. Mater. Sci.* **1996**, *6*, 15-50.
7. Kresse, G.; Furthmüller, J., Efficient Iterative Schemes for Ab Initio Total-Energy Calculations Using a Plane-Wave Basis Set. *Phys. Rev. B* **1996**, *54*, 11169.
8. Perdew, J. P.; Burke, K.; Ernzerhof, M., Generalized Gradient Approximation Made Simple. *Phys. Rev. Lett.* **1996**, *77*, 3865.
